# Case report: use of lenzilumab and tocilizumab for the treatment of coronavirus disease 2019

**DOI:** 10.2217/imt-2020-0136

**Published:** 2020-06-17

**Authors:** Megan Melody, Jared Nelson, Jacquelyn Hastings, Joshua Propst, Michael Smerina, Julio Mendez, Pramod Guru

**Affiliations:** ^1^Department of Internal Medicine, Mayo Clinic, Jacksonville, FL 32224, USA; ^2^Division of Infectious Disease, Mayo Clinic, Jacksonville, FL 32224, USA; ^3^Department of Critical Care Medicine, Mayo Clinic, Jacksonville, FL 32224, USA

**Keywords:** COVID-19, cytokine-release syndrome, GM-CSF, IL-6, inflammatory markers, lenzilumab, monoclonal antibody, pneumonia, severe acute respiratory distress syndrome, tocilizumab

## Abstract

**Background:** Coronavirus disease 2019 (COVID-19) is a novel disease associated with a cytokine-mediated, severe, acute respiratory syndrome. Tocilizumab and lenzilumab are recombinant monoclonal antibodies against IL-6 and granulocyte macrophage colony-stimulating factor, respectively, and have been proposed as a potential treatment for acute, hypoxic respiratory failure associated with COVID-19. **Results & methodology:** We present the case of a 68-year-old man with COVID-19 who was initially treated with hydroxychloroquine and lenzilumab, but continued to develop hypoxemia, requiring an increase in respiratory support with an associated rise in serum inflammatory markers. He was subsequently treated with tocilizumab with marked clinical improvement and a decrease in acute phase reactants within 48 h. **Discussion & conclusion:** This case demonstrates the effective use of tocilizumab in the treatment of COVID-19 and suggests the superiority of tocilizumab over lenzilumab in the management of this cytokine-mediated syndrome.

Severe acute respiratory syndrome coronavirus 2 (SARS-CoV-2) is the virus responsible for causing the coronavirus disease 2019 (COVID-19), which has become widely known due to its association with the development of acute hypoxic respiratory failure [1,2]. Despite nearly 4 million cases of COVID-19 being diagnosed worldwide, as of 9 May 2020, the literature has failed to identify any effective pharmacologic intervention [3–5]. Severe respiratory disease caused by COVID-19 is characterized by severe hypoxia and elevated serum levels of acute phase reactants including ferritin, C-reactive protein (CRP) and lactate dehydrogenase (LDH) [1,2,6,7]. Although the exact pathogenesis of this syndrome is unknown, it is thought to be due to high serum levels of pro-inflammatory mediators such as IL-6 [8,9]. Similar cytokine-release syndromes (CRS) are associated with toxicity from chimeric antigen receptor (CAR) T-cell therapy [10,11]. Tocilizumab is a humanized recombinant monoclonal antibody that acts as an IL-6 receptor antagonist and is effective at treating CRS associated with CAR T-cell therapies [12–14]. Lenzilumab, a recombinant monoclonal antibody against granulocyte macrophage colony-stimulating factor, has been shown to reduce several inflammatory mediators, making it another suggested treatment for CRS [15,16]. Given the similarities between CRS associated with CAR T-cell therapy and SARS-CoV-2, both lenzilumab and tocilizumab have been proposed as potential therapies to treat COVID-19 [17–20]. Tocilizumab is approved for a Phase III clinical trial for the treatment of COVID-19 and lenzilumab has been approved for COVID-19 patients under individual patient emergency Investigational New Drug applications [21–23]. Here we present a patient with SARS-CoV-2 pneumonia who was first treated with lenzilumab; however, he continued to decline and was then given tocilizumab with subsequent clinical and serologic improvement.

## Case description

A 68-year-old male presented to our institution with a 4-day history of productive cough, fever up to 39.3°C, nasal congestion, generalized weakness and progressively worsening dyspnea. He denied any other symptoms. Two weeks prior to presentation, patient returned from a hunting trip to New Zealand and was self-quarantined since that time. He denied any known COVID-19 exposures or other sick contacts.

The patient’s medical history was significant for hypertension, previously treated with an angiotensin-receptor blocker, but more recently managed with lifestyle modifications alone. Surgical history included bilateral inguinal hernia repair and tonsillectomy as a child. Social history was significant for moderate alcohol consumption and smoking two cigarettes daily.

**Figure 1.**
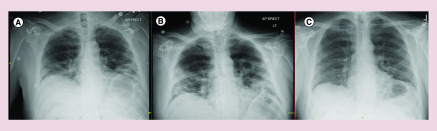
Radiographic images illustrating progression of COVID-19 related pneumonia. **(A)** Chest x-ray on day 4, prior to lenzilumab dosing, with bilateral, lower lobe predominant, parenchymal opacities. **(B)** Chest x-ray, prior to tocilizumab dosing, with worsening multifocal pneumonia. **(C)** Chest x-ray, 20 days post-tocililzumab dosing, with linear areas of scarring at prior sites of consolidation, consistent with healing COVID-19-related pneumonia. COVID-19: Coronavirus disease 2019.

In the emergency department, patient was febrile to 38.5°C, tachypneic with a respiratory rate of 33 and hypoxemic requiring 2–3 l of supplemental oxygen. Heart rate and blood pressure were normal. Pertinent examination findings included bibasilar crackles and increased work of breathing. Initial laboratory studies were significant for CRP of 61.2 mg/l (normal ≤8 mg/l), ferritin of 519 mcg/l (24–336 mcg/l), D-dimer of 571 ng/ml fibrinogen equivalent units (FEU; ≤500 ng/ml FEU) and an LDH of 282 U/l (122–222 U/l) (Table 1). His leukocyte count was normal at 4.0 (3.4–9.6 × 10^9^/l), with an absolute neutrophil count of 3.10 × 10^9^/l (1.56–6.45 × 10^9^/l). His renal function, transaminases and procalcitonin were normal. Initial chest x-ray demonstrated bilateral, lower lobe predominant, alveolar and interstitial opacities (Figure 1A). His nasopharyngeal swab was positive for COVID-19 PCR testing. ECG showed normal sinus rhythm with a QTc of 430 ms and glucose-6-phosphate dehydrogenase level was 7.9 U/g Hb (8.8–13.4 U/g Hb). Patient was started on oral hydroxychloroquine 400 mg for two doses, followed by 200 mg twice daily for an additional 4 days. Emergency Investigational New Drug approval was granted by the United States Food and Drug Administration for lenzilumab therapy; patient received a total of 3 doses of intravenous lenzilumab 600 mg in the initial 36 h of hospital admission. IL-6 testing from admission later returned at 37.1 pg/ml (<1.8 pg/ml). The remainder of his infectious workup, including blood cultures, nasopharyngeal swab PCR testing for influenza (A and B) and respiratory syncytial virus, *Streptococcus pneumoniae* urinary antigen and Legionella urinary antigen were negative.

**Table 1.** Acute phase reactants and O_2_ requirements over course of hospitalization.

	Day 4 presentation & lenzilumab dosing	Day 5	Day 6	Day 7	Day 8	Day 9	Day 10 tocilizumab dosing	Day 11	Day 12	Day 13	Day 14
WBC	4.0	4.0	5.5	5.0	6.5	5.7	6.1	5.0	4.0	4.6	4.6
ANC	3.10	–	4.51	3.62	4.85	4.80	4.42	3.11	2.21	–	–
CRP (mg/l)	44.9	61.2	83.9	82.8	86.5	152.0	175.8	174.7	145.7	63.6	–
LDH (U/l)	282	–	267	272	267	388	226	234	233	206	–
Ferritin (mcg/l)	519	–	611	–	736	–	745	–	842	–	–
Procalcitonin (ng/ml)	0.08	–	–	0.11	–	–	–	–	0.13	0.10	–
IL-6 (pg/ml)	27.1	34.2	30.8	30.9	95.4	–	57.6	363	–	**–**	125
FiO_2_ (%)	100	100	100	80	60	60	100	50	40	40	40
O_2_ (l/min)	3	2.5	4	50	50	50	60	50	30	30	30

ANC: Absolute neutrophil count; CRP: C-reactive protein; FIO_2_: Fraction of inspired oxygen; LDH: Lactate dehydrogenase; O_2_: Oxygen; WBC: White blood cell count.

**Figure 2.**
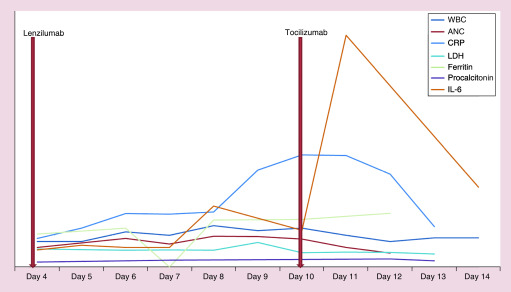
Trend of acute phase reactants over the patient’s hospital course. ANC: Absolute neutrophil count; CRP: C-reactive protein; LDH: Lactate dehydrogenase; WBC: White blood cell count.

Patient’s overall clinical condition remained stable, requiring 2–3 l of oxygen therapy by nasal cannula, until day 7 from symptom onset. At that time, he developed intermittent fevers and progressively worsening hypoxia. His worsening hypoxemic respiratory failure was managed by noninvasive ventilator methods including high flow nasal cannula and helmet positive pressure ventilation, intermittent prone positioning and fluid restriction. Repeat chest x-ray on day 10 was consistent with worsening multifocal pneumonia (Figure 1B). Laboratory studies revealed rising serum inflammatory markers including IL-6, ferritin, CRP and LDH (Figure 2). In addition, patient was noted to have a thrombocytosis, hyperfibrinogenemia and elevated D-dimer and was started on a therapeutic heparin drip and aspirin for suspected COVID-19 related hypercoagulable state. Due to clinical worsening and laboratory values suggestive of a hyperinflammatory cytokine surge, the decision was made to treat the patient with a single dose of iv. tocilizumab 680 mg (8 mg/kg) at 100 ml/h administered over 60 min, as per the institutional protocol.

Within 24 h of receiving tocilizumab, patient showed dramatic clinical improvement. He became afebrile, had significant decrease in oxygen requirements and his inflammatory markers showed a downward trend after 48 h (Figure 2). Given the patient’s overall improvement, further imaging and serum inflammatory markers were not obtained after 48 h following tocilizumab dosing. On day 15 following symptom onset, patient was weaned to standard nasal cannula. Follow-up COVID-19 PCR testing on days 15 and 16 were negative. There was no bleeding complications related to heparin and he was started on oral anticoagulation with a plan to finish four weeks of therapy. The patient was subsequently discharged from the hospital on day 17 with 2 l supplemental oxygen via nasal cannula. He was monitored via weekly video visits with continued improvement; he no longer required oxygen with exertion by day 26. Patient was seen in clinic on day 30 following initial symptom onset, at which time he remained without oxygen requirement and denied any shortness of breath, pleuritic chest pain or persistent cough. Laboratory studies showed a normal leukocyte count at 5.6 × 10^9^/l, absolute neutrophil count 2.86 × 10^9^/l, platelet count 225 × 10^9^/l (135–317 × 10^9^/l) and a CRP <3.0 mg/l (normal ≤8 mg/l). Hepatic function was also normal. Chest x-ray at the time of follow-up showed linear areas of scarring in the mid lower lung zones at the sites of prior airspace consolidation, consistent with healing COVID-19-related pneumonia.

## Materials & methods

For analysis, we reviewed patient’s electronic medical record which included clinician notes, laboratory tests, microbiology results and imaging. Per institutional guidelines, this case report was exempt from Institutional Review Board review. Verbal and written patient consent was obtained prior to preparation of this case.

## Discussion

We present a patient who initially presented to our institution with COVID-19-related pneumonia and had clinical progression of symptoms despite receiving three doses of lenzilumab and oral hydroxychloroquine. Due to worsening respiratory status, patient was given tocilizumab with rapid clinical improvement. Prior to administration of tocilizumab, there was concern regarding the safety of using two monoclonal antibodies that inhibit different targets of the same T-cell activation pathway; however, the patient was able to tolerate both medications. The half-life of tocilizumab is 11–13 days and the most common adverse events associated with tocilizumab are gastrointestinal perforation, neutropenia, thrombocytopenia, hepatic injury or hyperlipidemia [24]. Patient was seen in clinic for scheduled follow-up 20 days post-tocilizumab dosing. At that time, patient had normal hepatic function with a normal leukocyte count and platelet count. Lipid profile was not obtained at the time of follow-up. Although further monitoring is needed to assess for long term side effects of tocilizumab, initial follow-up suggests no significant adverse reaction in the setting of dual monoclonal antibody administration.

Although patient’s significant clinical improvement coincides with timing of tocilizumab dosing, tocilizumab was dosed within 5 days of last lenzilumab dose. The timing of the dosing of these medications makes it unclear if significant clinical improvement can be attributed to tocilizumab in isolation, or whether it resulted from the concomitant dosing of these two medications.

This case describes a patient with significant clinical decline at day 10 after symptom onset with increasing oxygen requirements and increasing serum levels of acute phase reactants. This time course coincides with the literature, which cites that most patients require escalation of care or max severity of symptoms between 9–11 days after symptom onset [25,26]. Therefore, patient’s clinical improvement on day 11 and 12 does not match with standard disease trajectory and is most likely attributed to medical intervention with administration of tocilizumab.

## Conclusion

To our knowledge, this is the first case reported in the literature in which both lenzilumab and tocilizumab have been used in the same patient for the treatment of COVID-19 and sparks the discussion regarding the safety of subsequent use of the two monoclonal antibodies. In addition, this case demonstrates the effective use of tocilizumab for the treatment of SARS-CoV-2 and suggests the superiority of tocilizumab over lenzilumab in the management of this cytokine-mediated syndrome. Further investigation is needed through the approved Phase III clinical trial with tocilizumab and the lenzilumab trial.

**Summary points**

- Coronavirus disease 2019 (COVID-19) is associated with the development of a cytokine mediated severe, acute, hypoxic, respiratory failure.
- To date, no current therapy has proven effective in the literature for the management of this syndrome.
- Tocilizumab is an IL-6 receptor antagonist that has been effective in a cytokine-mediated syndrome associated with chimeric antigen receptor T-cell therapy.
- Lenzilumab, a recombinant monoclonal antibody against granulocyte macrophage colony-stimulating factor, has been shown to reduce several inflammatory mediators.
- We present a case of 68-year-old male who developed severe, acute, respiratory failure in the setting of COVID-19.
- The patient was treated with hydroxychloroquine and lenzilumab with continued respiratory decline and associated increase C-reactive protein, ferritin, D-dimer and lactate dehydrogenase.
- Within 48 h of tocilizumab dosing patient had marked clinical improvement and subsequent decline in serum inflammatory markers.
- To our knowledge, this is the first case reported in the literature in which both lenzilumab and tocilizumab have been used in the same patient for the treatment of COVID-19.
- This case report sparks a discussion regarding the use of tocilizumab versus lenzilumab in the management of the current worldwide pandemic.
